# Testing Industry Assumptions About Raters’ Clinical Experience and Performance During Alzheimer’s Disease Clinical Trials

**DOI:** 10.1002/alz70861_108900

**Published:** 2025-12-23

**Authors:** Katrina Patrick, Meagan T. Farrell, Cristian Sirbu, Cynthia W McNamara

**Affiliations:** ^1^ Cronos Clinical Consulting Services, Inc. an IQVIA business, New Providence, NJ USA; ^2^ IQVIA Patient Centered Solutions, New York, NY USA

## Abstract

**Background:**

Standardizing implementation of complex clinical outcome assessments (COAs) in Alzheimer’s disease (AD) clinical trials requires trained and qualified site raters. Best practices include setting minimum education and experience criteria, along with robust training programs. However, there is significant variability in the level of rigor applied to this process, often due to timeline and resource limitations. This study aimed to investigate the relation between raters’ experience and training performance and their in‐trial rater performance, as defined by remediations required during the trial.

**Method:**

Data were analyzed from 160 raters across four AD clinical trials, including three countries and 70 sites. During startup, raters were categorized as fully qualified (via education, experience, and performance on applied scoring exercise) or sponsor exception (missing criteria but exempted into trial). Logistic regression was used to explore predictors of rater in‐trial performance (remediation during trial vs. no remediation). Predictors included 1) remediations during training, 2) rater qualification status, 3) AD clinical experience, and 4) AD research trial experience.

**Result:**

Thirty‐eight (23.7%) of raters required remediation during trial. The average AD clinical experience was 108 months (SD=78 months), and average AD trials experience was 71 months (SD=52 months). Overall, the logistic regression model was significant c^2^ (4) = 20.6, *p* <.01, and remediation during training (B=1.91, SE=0.59, Wald=10.55, *p* <.001) and AD clinical experience (B= ‐0.01, SE=0.006, Wald = 6.68, *p* <.01) were significant predictors of remediation during trials.

**Conclusion:**

These data confirm the importance of AD‐specific clinical experience and performance during pre‐trial training exercises as key indicators of rater performance during clinical trials. Sponsors should consider performance during pre‐trial startup activities when selecting raters to ensure that COAs are accurately scored and reliably implemented throughout the duration of the trial. Planned analyses will explore additional predictors of rater performance (e.g., individual and site level factors), as well as the implications of rater remediation on trial outcome data (i.e., impacts on data reliability).

